# Hygiene

**Published:** 1880-06

**Authors:** 


HYGIENE.—We were glad to see that the able Baltimore Correspondent of the *Boston Medical and Surgical Journal*, foreshadowed in his communication in the issue of May 13th ultimo, what has been actually done by us, in adding the department of *Hygiene*, as one of the subjects appertaining to medical science, that will also claim our attention in future issues of the journal. It is our purpose to make our serial as acceptable and useful as possible, to all who have, and may hereafter, honor us with their patronage and encouragement and to render it a full equivalent for the price of its annual subscription; we would, therefore, respectfully solicit contributions from gentlemen engaged in the investigation of sanitary science, so that our new department may be enriched by such recent discoveries and suggestions as may be made therein.

